# Effect of human beta-defensin-3 on head and neck cancer cell migration using micro-fabricated cell islands

**DOI:** 10.1186/1758-3284-4-41

**Published:** 2012-06-28

**Authors:** Kevin Wang, Joanne H Wang, Harihara Baskaran, Russell Wang, Rick Jurevic

**Affiliations:** 1 College of Medicine, University of Cincinnati, Cincinnati, OH 45267, USA; 2 Warren Alpert Medical School, Brown University, Providence, RI, 02912, USA; 3 Department of Chemical Engineering, Case Western Reserve University School of engineering, Cleveland, OH 44106, USA; 4 Department of Comprehensive Care, Case Western Reserve University School of Dental Medicine, Cleveland, OH 44106, USA; 5 Department of Biological Sciences, Case Western Reserve University School of Dental Medicine, Cleveland, OH 44106, USA

**Keywords:** Head and neck cancer, Cancer cell migration, Human beta defensin, Migration index, Micro-fabricated cell islands

## Abstract

**Background:**

To examine the effect of the natural antimicrobial peptide human β-defensin-3 (hBD-3), on the migration of a head and neck cancer cell line *in vitro* using microfabrication and soft-lithographic techniques.

**Methods:**

TR146 cancer cells were seeded in Petri dishes with microfabricated wells for cell migration assays. Total 54 cell islands were used of various shape and size and experimental media type. Cell migration assays were analyzed in six group media: Dulbecco’s modified medium (DMEM); DMEM with vascular endothelial growth factor (VEGF); Conditioned media of human embryonic kidney cells (HEK 239) expressing hBD-3 via transfected cloned pcDNA3 as CM/hBD-3; CM/hBD-3 + VEGF; conditioned medium from non-transfected HEK 239 (not expressing hBD-3) as control (CM); and the last group was CM + VEGF. Cell islands were circular or square and varied in size (0.25 mm^2^, 0.125 mm^2^, and 0.0625 mm^2^). Cell islands were imaged at t = 0 h, 3 h, 6 h, and 24 h.

**Results:**

The results show cancer cell islands that originally were smaller had higher migration indices. There was no difference of MIs between circular and square cell islands. MIs at the end point were significantly different among the groups except between CM and CM-hBD-3+ VEGF.

**Conclusions:**

VEGF enhanced cancer cell migration. The combination of DMEM and VEGF showed a synergistic effect on this phenomenon of cancer cell migration. Conditioned medium with hBD-3 suppressed cancer cell migration. hBD-3 suppressed VEGF enhancement of TR146 cancer cell migration.

## Background

In the head and neck region, two of the most common types of cancer are cancer of the oral cavity and cancer of the oropharynx. More than 35,000 Americans are diagnosed each year with oral or oropharyngeal cancer, which will cause over 8,000 deaths, killing roughly one person per hour. Ninety to ninety-five percent of those cancers are squamous cell carcinomas [1].

Cancer metastasis to vital organs is the main cause of mortality in cancer patients. Growth rate and metastatic potential in different types of cancer cells are important clinically for both selection of treatment and for prognosis. The metastatic potential of a cancer cell is related not only to the complex biological cascade of autocrine, paracrine, and endocrine signaling processes of cell-cell and cell-substrate interactions, but also to inherited ability of cell movement [2]. There are many signaling pathways involved in cell migration, including actin and microtubule dynamics, cell-cell and cell-extracellular matrix adhesion, and intracellular trafficking of proteins [3]. Preventing the process of metastasis, the spread of cancer cells from a primary tumor to a secondary site, is cardinal therapeutic approach for treating cancer patients.

In the 1980s, endogenous antimicrobial peptides (AMPs) were found at high concentrations in the granules of neutrophils and macrophages. These antimicrobial peptides were named “defensins”. Human β-defensin (hBD) expression has been identified in many tissues, almost uniformly in the epithelium of the oral cavity. Specifically, human beta defensin-3 (hBD-3) is a small, cationic peptide that primarily is known for its antimicrobial, antifungal, and antiviral properties [4]. β-defensins, initially identified as broad-spectrum antimicrobial peptides, increasingly have been observed to exhibit numerous other *in vitro* and *in vivo* activities that do not always relate directly to host defense. In addition to direct antimicrobial activity, β-defensins exhibit potent chemotactic activity for a variety of innate immune cells and stimulate other cells to secrete cytokines [5].

The expression of β-defensins in cancer is controversial for example; diminished hBD-1 expression has been reported for renal and prostate cancer [6,7]and for basal cell carcinoma [8]. Loss of expression of hBD-1, hBD-2, and hBD-3 in oral squamous cell carcinoma has also been reported [9]. In contrast, elevated hBD-1 expression has been reported to occur within renal cell carcinomas [10]. Lung cancer patients have elevated hBD-1 in their serum, along with upregulated hBD-2 [11]. Human β-definsin-3 expression has been shown to be increased in vulvar squamous cell carcinoma [12]. Data on β-defensin expression in oral squamous cell carcinoma (OSCC) also are in conflict. Low levels of hBD-2 expression in OSCC have been linked to poor differentiation. In contrast, other studies have reported increased hBD-2 expression in OSCC compared with normal epithelial cells [13,14].

hBD-3 is chemotactic for immature dendritic cells, memory T cells, and mast cells. There is increasing evidence that human β-defensins are differentially regulated in cancers such as OSCC. Overexpression of hBD-3, but not hBD-1 and hBD-2, has been shown in pre-malignant cells in carcinoma in situ lesions. hBD-2 is associated with tumor-associated macrophage (TAM) trafficking in oral cancer [15]. The function of overexpression of hBD-3 in carcinoma in situ and in malignant cells is unclear and its contribution to cancer cell migration is unknown. Novel anticancer agents are needed when resistance exists against conventional chemotherapy. Natural antimicrobial peptides or synthetic derivatives may be used as novel strategies against neoplastic growth and may represent a novel family of anticancer agents. However, future research is needed to understand the role of antimicrobial peptides in cancer and to develop potential anticancer drugs.

Currently, the most relevant methods of determining the metastatic potential of neoplasia are *in vivo* assays involving tumor cell implantation in immunodeficient animals. These methods, however, are expensive and the results may depend on the site or route of cancer cell entry. Use of *in vitro* methodology to predict the metastatic potential of cancer cells can be useful for predicting those cancer cell behaviors *in vivo*. At present, common methods of *in vitro* models of tumor cell invasion use Mitrgel assays which have a filter in between two chambers. Tumor cells are dispersed in the upper chamber while chemotaxants are dispersed in the lower chamber. A barrier is removed and the cells migrate over a set amount of time through the gel, which is then stained and the tumor cells counted. The drawback of this method is that the observation time is randomly selected and the concentration of the chemotactic agents diffusing into the gel may not consistent as a function of time over a multiple experiments [16].

Microfabrication combined with soft lithographic methods is a useful platform for patterning proteins and cells. Soft lithography refers to a family of techniques for fabricating or replicating structures using “elastomeric stamps, molds, and conformable photomasks.” Elastomeric materials are used, most notably Polydimethylsiloxane (PDMS). PDMS has several properties that make it suited for patterning proteins and cells. PDMS is biocompatible, is permeable to gases, and is used in cell cultures. It is optically transparent. Because it is elastomeric, it conforms to non-planar surfaces well. Soft lithography that is derived from photolithography and associated techniques which offers tools for micropatterning that complement and extend conventional fabrication methods for biological research [17]. By using these microfabrication and soft lithography techniques; we examined the effect of a natural antimicrobial peptide, human β-defensin-3, on a human head and neck cell line (TR146) migration *in vitro*.

## Methods

### Cancer cell culture

TR146 cell line is a metastatic human buccal tumor cell line isolated from the neck. The TR146 cell line was maintained at 5% CO^2^; 37°C in Dulbecco’s modified media (DMEM) with 10% fetal bovine serum (Invitrogen, Carlsbad, CA) and 100 IU/mL penicillin, 100μg/mL streptomycin, and 0.25μg/mL amphotericin B. The cultures were split at sub-confluence (70-80%) 1:4 seeding at 3x10^4^cells/cm^2^.

### Human β-Defensin-3

Human embryonic kidney 293 cells (HEK293; ATCC, Manassas, VA) were maintained in Dulbecco’s modified media (DMEM) supplemented with 10% fetal bovine serum (Invitrogen, Carlsbad, CA) and antibiotics (100 IU/mL penicillin, 100μg/mL streptomycin, 0.25μg/mL amphotericin B). To generate a stable HEK 293 cell line expressing hBD-3, the coding region of hBD-3 was cloned into pcDNA3 (Invitroge) and transfected into HEK293 cells using Lipofectamine 2000 following the manufacturer’s protocol (Invitrogen) [18]. HEK293 cells over-expressing HBD-3 (termed HEK/hBD-3) were subjected to hBD-3 enzyme-linked immunosorbent assay (ELISA) analysis to determine hBD-3 secretion and concentration [19]. To prepare conditioned media of hBD-3, HEK/hBD-3 were cultured in DMEM 10%FBS overnight and then the cells were cultured in serum-free DMEM for 24 h. The medium (CM/hBD-3) was collected and centrifuged at 5,000x*g* to remove cell debris and again the concentration of hBD-3 was determine by ELISA and solution was diluted to 20 ng/mL. 3 ml of the conditioned medium with 20 ng of hBD-3 was used as the CM/hBD-3 solution. Conditioned media derived from HEK293 cells without hBD-3 transfection was used as controls (CM).

### Fabrication of PDMS membranes by soft lithography containing microarrays

Polydimethylsiloxane (PDMS) membranes were obtained using a standard combination of photolithography and soft lithography techniques [17]. AutoCAD (Autodesk, Inc., San Rafael, CA) microarrays were used to obtain photomasks (Advance Reproductions, North Andover, MA). The array patterns on a silicon wafers were spin-coated with a negative photoresist SU-8 2075 (Microchem Corp, Newton, MA). The photoresist was then processed with the photomask to produce a negative template. The polymer PDMS (Sylgard 184, Dow Corning, Midland, MI) was used to create a positive template by spin-coating and curing at 80 ^0^C. The resulting membranes were about 100 μm thick. The membranes were attached to a PDMS ring (60 mm inner diameter, 4 mm thick) for easy handling and stability.

### Development of cell islands

**Figure 1:**
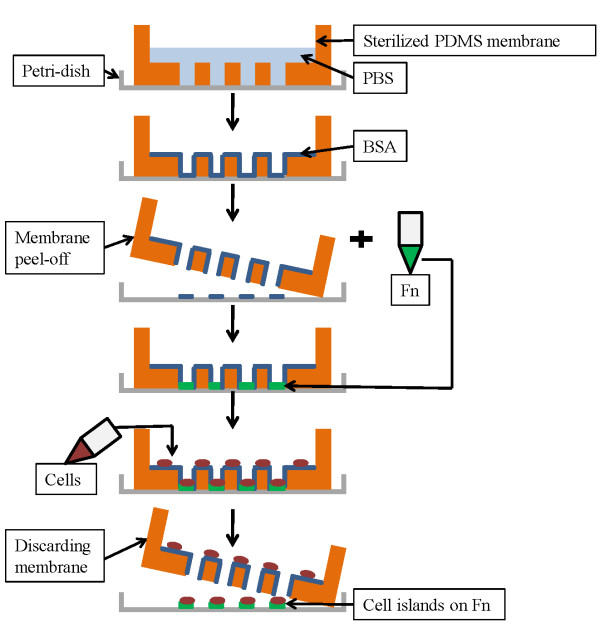
Steps for cell island fabrication using PDMS membranes.

Individual square or circular cell islands of three sizes (0.25 mm^2^, 0.125 mm^2^, and 0.0625 mm^2^) were arranged into microarrays in the PDMS membrane. The thickness of the PDMS membrane was approximately 100 μm. A PDMS ring 4 mm thick was attached to the membrane to enable safe handling and to create wells in which the suspended cells were deposited. The membranes were sterilized in 100% ethanol (Fisher Scientific, Pittsburgh, PA) overnight, then transferred to a fresh 50 mm Petri-dish (BD, Franklin Lakes, NJ) and allowed to adhere to the dish surface. Two milliliters of 1X phosphate buffered saline (PBS) were pipetted into the well and placed under vacuum for one hour. The PBS was aspirated and was replaced with 1% w/v BSA in 1x PBS for 30 min at room temperature in a sterile environment and then aspirated. The BSA prevents the cells from adhering to the PDMS surface. Figure 1 is a schematic of cell island development using PDMS membrane.

Fibronectin (20 mg/mL in PBS) was added and incubated onto the cell-contact surfaces for 1 h at 37 ^0^ C and 5% CO_2_. The fibronectin (FN) solution was aspirated and 1.0x 10^6^ TR146 cells were seeded on the membrane and allowed to attach overnight in order to form confluent circular or square “cell islands” with areas of 0.25 mm^2^, 0.125 mm^2^, and 0.0625 mm^2^ each. The PDMS membranes were then removed and cell islands gently washed with phosphate-buffered saline (PBS, Invitrogen). Two milliliters of fibronectin were added and the Petri dishes incubated for 1 h of 37 ^0^ C and 5% CO_2_. The fibronectin solution was then aspirated and experimental media was added to the cells. Cell migration studies also were conducted without the use of fibronectin. In the experiments without fibronectin, only DMEM was used as the experimental media.

### Cell migration assay

Two-dimensional cell migration from well-formed consisting of confluent cell islands was studied in 60 mm Petri dishes for 24 h since the doubling time for TR 146 cells in culture is 1.5 day. Islands were imaged at t = 0 h (initial time point) and at t = 3, 6, 12, and 24 h (end time point). We chose a 24-h end point because this represented the optimal time that minimized cell proliferation effect on island expansion.

Phase contrast images of the cells islands were taken with an Olympus IX71 inverted microscope (Olympus, Japan) using an UPlanFl 10x objective and the software ImagePro (Media Cybernetics, Bethesda, MD).

TR146 cancer cell island migration was studied under different solution environments. Six groups were designed in this study: Dulbecco’s modified medium (DMEM); DMEM with vascular endothelial growth factor (VEGF); Conditioned media of human embryonic kidney cells (HEK 239) expressing hBD-3 via transfected cloned pcDNA3 (CM/hBD-3); CM/hBD-3 + VEGF; conditioned medium from non-transfected HEK 239 (not expressing hBD-3) as control (CM); and CM + VEGF.

### Analyzing cell islands

Phase contrast images of each initial cell island area and the areas from the same cell island at different time intervals were analyzed. Image J (Rasband WS, ImageJ, U. S. National Institutes of Health, Bethesda, MD, USA, http://rsb.info.nih.gov/ij) was used to quantify the cell island areas. The area occupied by each cell island was measured immediately after the removal of PDMS membrane (t = 0 h) and then after 3 h, 6 h, and 24 h of migration. Migration was quantified as the area of island at any given time normalized to the initial area, with margins of cell islands tracked manually. This parameter was termed Migration Index (MI). It is a more accurate way of quantifying migration from cell islands because it takes into account the initial size differences of the islands, allowing us to compare migration of various sizes and shapes of islands. The Migration Index was expressed as mean ± standard deviation. Experiments were performed in triplicate and at least 9 cell islands (n = 9) in total were used for each cell island shape and size and experimental media type. The migration characteristics of islands of these cells were studied *in vitro* by monitoring their migratory behavior.

### Statistical analysis

Cell migration assays were averaged across replicate experiments. Statistical analyses of the data were performed by analysis of variance (ANOVA) to examine the general trends, and individual comparisons were performed by using Tukey’s test. A p-value of <0.05 was considered as statistically significant. Three variables were analyzed at different time intervals: 3 h, 6 h, and 24 h. The three variables are original cell island sizes (0.25 mm^2^, 0.125 mm^2^, and 0.0625 mm^2^), cell island shapes (square and circle), and the six different experimental media.

## Results and discussion

Every effort was made to develop uniform monolayer cell islands. A cell density distribution curve was generated with square islands. Average cell count correlate linearly with island size, thus indicating that consistent cell density was maintained for all islands.

**Figure 2:**
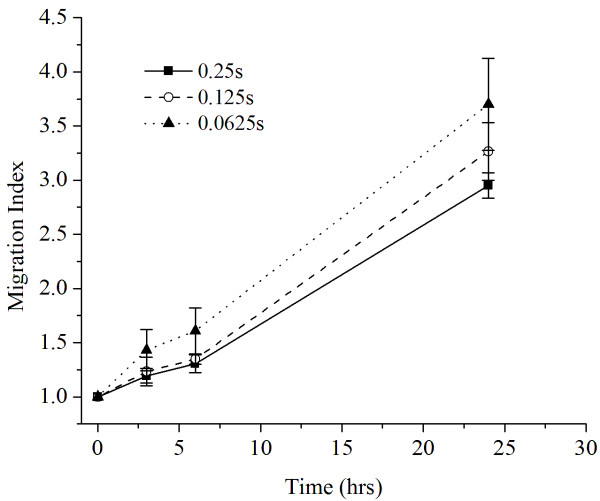
**Migration indices of square cell islands (0.0625, 0.125, and 0.25 mm**^**2**^**) in DMEM medium as function of time.**

Figure 2 shows smaller cell islands had significantly larger MI (p < 0.01) compared to the larger islands at 3 h, 6 h, and 24 h points. The smallest square island (0.0625 mm^2^) showed an average MI of 1.60 ± 0.21 in DMEM environment while the largest square island (0.25 mm^2^) showed an MI of 1.30 ± 0.09 after 6 h. When comparing 24 h results in the same medium, MI was 3.71 ± 0.42 for the smallest square island category while the largest square island showed a Migration Index of 2.95 ± 0.37. They were statistically different at the three time intervals.

**Figure 3:**
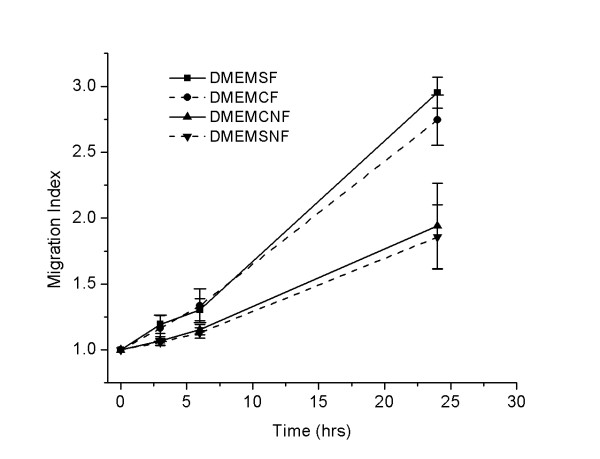
**Cell migration with and without fibronectin using square and circle cell islands at different time intervals.** DMEMSF = square cell islands in DMEM, treated with fibronectin; DMEMCF = circular cell islands in DMEM, treated with fibronectin; DMEMSNF = square cell islands in DMEM without fibronectin treatment; DMEMCNF = circular cell islands in DMEM without fibronectin treatment. Cell island size was 0.25 mm^2^.

Figure 3 shows the migration index of 0.25 mm^2^ circle and square islands with and without fibronectin. There was no statistical difference between the Migration Indices of the two shape design, circle vs. square in the same sized category. The p-values of the difference of the MI between the circle and square cell islands with fibronectin at 3, 6, and 24 h were 0.885, 0.8888, and 0.866 respectively. The p-values of the difference of the MI between the circle and square islands without fibronectin at 3, 6, and 24 h were 0.8786, 0.88491, and 0.8759 respectively. However, there was a significant difference between with and without fibronectin groups. Only 0.25 mm^2^ circle cell island data were analyzed for comparison of the effect of different media on cancer cell migration.

**Figure 4:**
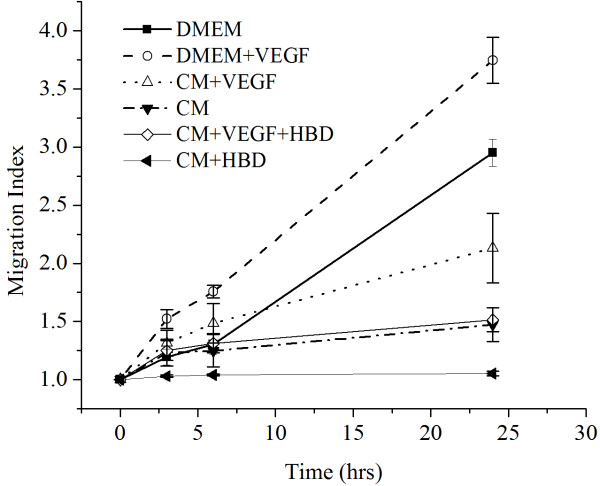
**Migration indices of the 0.25 mm**^**2 **^**cell islands in the different experimental media.**

**Figure 5:**
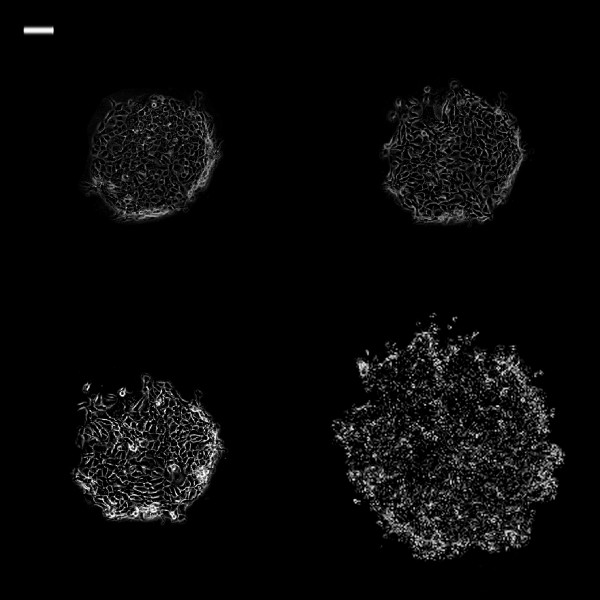
**Phase contrast images of 0.25 mm**^**2 **^**TR146 cell islands in DMEM at different time intervals.** White bar on the upper left is 100 μm. (Top left: 0 h; top right: 3 h; bottom left: 6 h; bottom right: 24 h).

**Figure 6:**
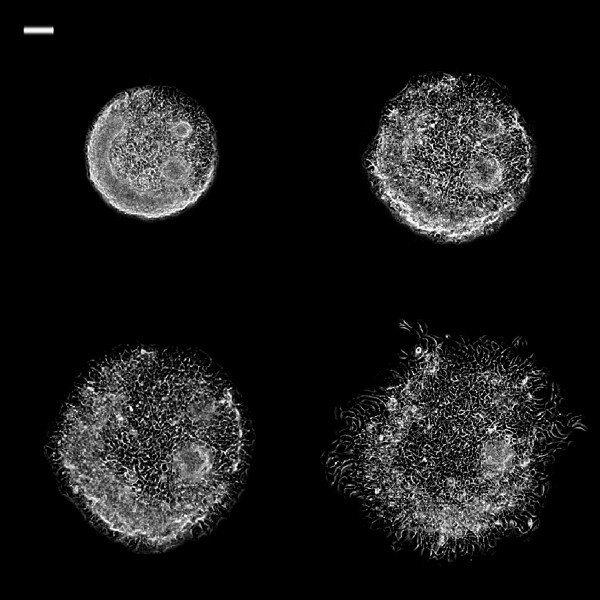
**Phase contrast images of 0.25 mm**^**2 **^**TR146 cell islands in DMEM + VEGF at different time intervals.** Phase contrast images of 0.25 mm^2^ TR146 cell islands in DMEM + VEGF at different time intervals. White bar on the upper left is 100 μm. (Top left: 0 h; top right: 3 h; bottom left: 6 h; bottom right: 24 h)(Top left: 0 h; top right: 3 h; bottom left: 6 h; bottom right: 24 h).

**Figure 7:**
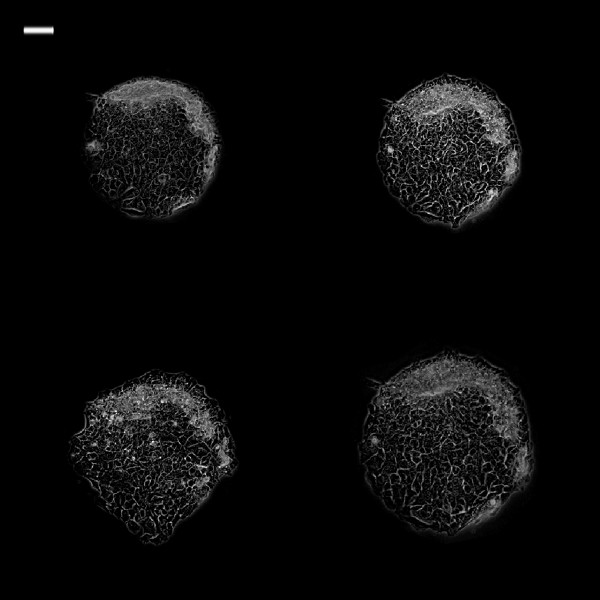
**Phase contrast images of 0.25 mm**^**2 **^**TR146 cell islands in CM at different time intervals.** White bar on the upper left is 100 μm. (Top left: 0 h; top right: 3 h; bottom left: 6 h; bottom right: 24 h).

**Figure 8:**
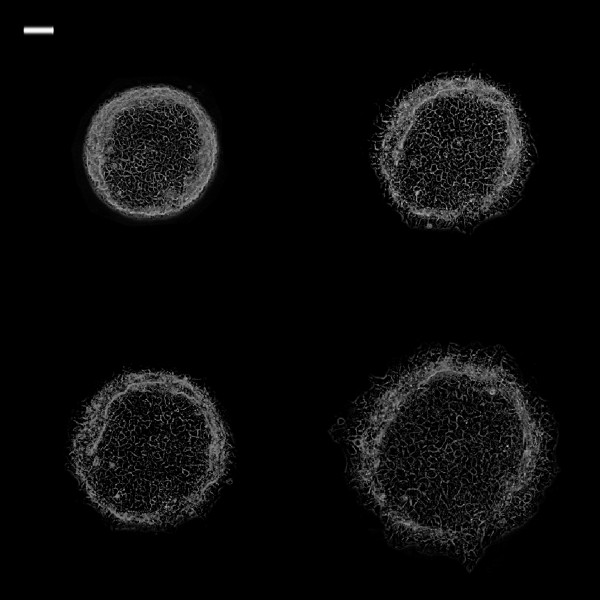
**Phase contrast images of 0.25 mm2 TR146 cell islands in CM + VEGF at different time intervals.** White bar on the upper left is 100 μm. (Top left: 0 h; top right: 3 h; bottom left: 6 h; bottom right: 24 h).

**Figure 9:**
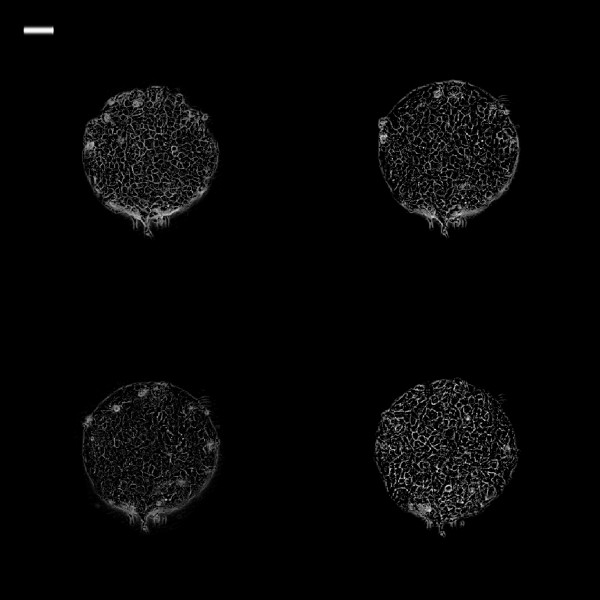
**Phase contrast images of 0.25 mm**^**2 **^**TR146 cell islands in CM + hBD at different time intervals.** White bar on the upper left is 100 μm. (Top left: 0 h; top right: 3 h; bottom left: 6 h; bottom right: 24 h).

**Figure 10:**
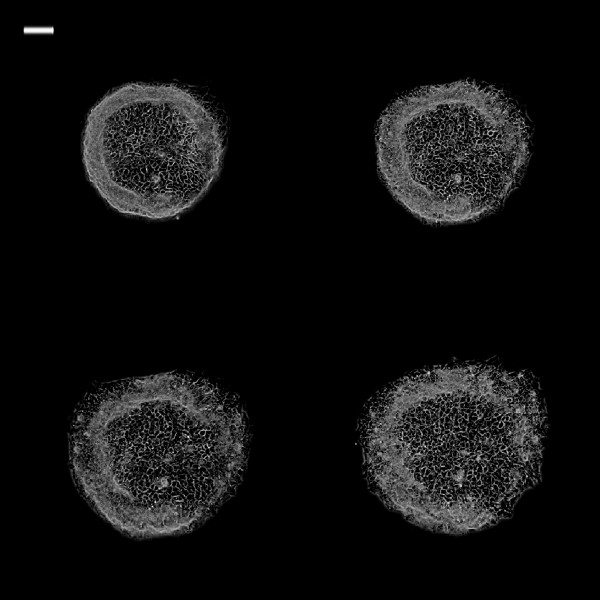
**Phase contrast images of 0.25 mm**^**2 **^**TR146 cell islands in CM + VEGF + hBD at different time intervals.** White bar on the upper left is 100 μm. (Top left: 0 h; top right: 3 h; bottom left: 6 h; bottom right: 24 h).

Figure 4 is a graph showing the different cell migration profiles in different conditions. DMEM + VEGF group had the highest MI followed by DMEM group. CM/HBD-3 had the lowest migration index and was statistically different from all the other groups (P ≤ 0.01). Figures 5, 6, 7, 8, 9 and 10 are typical micro-images of cancer cell migration of 6 groups as function of time and condition.

The *in vitro* microfabrication and soft lithography-based migration assays for this experiment allowed for the accomplishment of the following: (a) the inclusion of different patterns of microarrays within the same stencil, (b) the ability to generate behavior of rapid migration of cells and (c) ease of use and analysis. This migration model was successfully used to investigate migration of a TR146 cancer cell line under various experimental conditions. The model would be used for future investigation to quantify the effect of heterotypic interaction of the TR146 cell islands with other cells.

The larger cell islands depict a greater final area compared the smaller islands, but this must be studied in the proper context, *i.e.*, these larger islands already had a much larger initial area compared to the smaller islands. However, interestingly, the MI of smaller TR146 cell islands is higher compared to larger cell islands. The lower MI of larger cell islands can be due to local cell-cell interactions between the TR146 cells, inhibiting migration behavior within the cell islands. These *in vitro* results may reflect the behavior of TR146 tumors clinically. It is possible that tumor size may have a strong impact on metastatic migration of cancer cells. However, the TR146 cell line may also behave differently in an *in vitro* monolayer- and filter-based assays environment compared to the more three-dimensional *in vitro* environment. Clearly, significant future investigation will needed to resolve these questions.

The MI was used compare migration across island sizes and shapes, as it normalizes each area to its initial value. Circle and square islands are different in shapes and perimeters. The effect of shape was quantified by calculating the migration indices of islands of each shape at 3 time intervals. While circular and square-shaped islands retained the effect of size, *i.e.*, increasing MI with decreasing island sizes, there was no significant difference in migration between circle and square islands whether they were in the presence or absence of fibronectin. These results indicate that amount of migration is independent relative to the cell island shape, which can allow flexibility in future experimental designs in using this type of migration model.

The standard deviations of cell MI were large with small cell islands, therefore, we chosen to use large size with circle shape islands to analyze the effect of different environments on cancer cell migration.

When the cells were exposed to DMEM with or without fibronectin, the cells without fibronectin migrated less than cells with fibronectin, which was expected, as fibronectin is an important molecule in extracellular matrix that enhances cell adhesion and migration. The TR146 cell islands were established on FN-coated surfaces which enhance cell-cell adhesion inside the cell island and simulate an extracellular environment outside the cell island area. From the baseline area measurements of the media containing DMEM and CM (conditioned media); the addition of VEGF increased the migration rates of the cells. VEGF is known to stimulate tumor angiogenesis *in vivo* and thereby to facilitate tumor growth and metastases. VEGF has been shown to stimulate endothelial cell mitogenesis and cell migration, as well as other non-neoplastic cell types. VEGF concentration in the tumor tissues showed a relationship with the clinical stage and histologic grade of the tumor [20].

Interestingly, the most amount of migration occurred with DMEM + VEGF, even more than CM + VEGF, which implies some migratory inhibition factors present in CM that prevent maximal potential migration of the cells when exposed to VEGF. Further studies will be needed to determine if there are other factors are in the CM from HEK 239 that inhibits migration.

With the conditioned media from HEK239 cells of over-expressed hBD-3, migration rates were markedly decreased in both media with and without VEGF compared to CM with and without VEGF, respectively. CM/hBD-3 resulted minimal migration, while CM/hBD-3 + VEGF resulted migration levels that were equivalent to CM alone. This implies a possible inhibitory effect by hBD-3 on the VEGF pathway that promotes cell migration.

## Conclusions

The platform technology we have developed for this study provides advantages to continuously measure cancer cell migration as a function of time. The migration of TR 146 cancer cell islands is strongly dependent on island sizes, but not on island shapes. TR146 cell migration was promoted by both fibronectin and VEGF. Cancer cell migration was inhibited in all media containing hBD-3 compared to all respective media without hBD-3, including VEGF, a known potent stimulator of cell migration.

## Abbreviations

hBD: Human β-defensin; AMPs: Endogenous antimicrobial peptides; PDMS: Polydimethylsiloxane; DMEM: Dulbecco’s modified medium; HEK 239: Human embryonic kidney cells line 239; FN: Fibronectin; CM: Conditioned media; VEGF: Vascular endothelial growth factor; MI: Migration index.

## Competing interests

The authors declare that they have no competing interests.

## Authors’ contributions

KW, data acquisition, data analysis, writing manuscript, critical review of manuscript, approval; JW, data acquisition, statistical analysis, critical review of manuscript, approval; HB, concept and design, critical review of manuscript, approval; RW, concept and design, writing manuscript, critical review of manuscript, approval; RJ, critical review of manuscript, approval. All authors read and approved the final manuscript.

## Author information

KW, School of Medicine, University of Cincinnati, Cincinnati, OH, JW, Warren Alpert School of Medicine, Brown University, Providence, RI, HB, Department of chemical Engineering, RW. Department of Comprehensive Care, RJ, Department of Biological Sciences, Case Western Reserve University Cleveland, OH.
